# Risk of ischemic stroke and the use of individual non-steroidal anti-inflammatory drugs: A multi-country European database study within the SOS Project

**DOI:** 10.1371/journal.pone.0203362

**Published:** 2018-09-19

**Authors:** Tania Schink, Bianca Kollhorst, Cristina Varas Lorenzo, Andrea Arfè, Ron Herings, Silvia Lucchi, Silvana Romio, René Schade, Martijn J. Schuemie, Huub Straatman, Vera Valkhoff, Marco Villa, Miriam Sturkenboom, Edeltraut Garbe

**Affiliations:** 1 Leibniz Institute for Prevention Research and Epidemiology – BIPS, Bremen, Germany; 2 RTI Health Solutions, Barcelona, Spain; 3 University Milano-Bicocca, Milano, Italy; 4 PHARMO Institute, Utrecht, The Netherlands; 5 Local Health Authority ASL Cremona, Cremona, Italy; 6 Erasmus University Medical Center, Rotterdam, The Netherlands; Universita degli Studi di Napoli Federico II, ITALY

## Abstract

**Background and purpose:**

A multi-country European study using data from six healthcare databases from four countries was performed to evaluate in a large study population (>32 million) the risk of ischemic stroke (IS) associated with individual NSAIDs and to assess the impact of risk factors of IS and co-medication.

**Methods:**

Case-control study nested in a cohort of new NSAID users. For each case, up to 100 sex- and age-matched controls were selected and confounder-adjusted odds ratios for current use of individual NSAIDs compared to past use calculated.

**Results:**

49,170 cases of IS were observed among 4,593,778 new NSAID users. Use of coxibs (odds ratio 1.08, 95%-confidence interval 1.02–1.15) and use of traditional NSAIDs (1.16, 1.12–1.19) were associated with an increased risk of IS. Among 32 individual NSAIDs evaluated, the highest significant risk of IS was observed for ketorolac (1.46, 1.19–1.78), but significantly increased risks (in decreasing order) were also found for diclofenac, indomethacin, rofecoxib, ibuprofen, nimesulide, diclofenac with misoprostol, and piroxicam. IS risk associated with NSAID use was generally higher in persons of younger age, males, and those with a prior history of IS.

**Conclusions:**

Risk of IS differs between individual NSAIDs and appears to be higher in patients with a prior history of IS or transient ischemic attack (TIA), in younger or male patients. Co-medication with aspirin, other antiplatelets or anticoagulants might mitigate this risk. The small to moderate observed risk increase (by 13–46%) associated with NSAIDs use represents a public health concern due to widespread NSAID usage.

## Introduction

Non-steroidal anti-inflammatory drugs (NSAIDs) are frequently used medicines. Traditional NSAIDs (tNSAIDs) are associated with a 3- to 5-fold increased risk of serious upper gastrointestinal complications which is about 50% lower with the use of COX-2 selective inhibitors (coxibs).[1–3] Concerns about the cardiovascular (CV) safety were first raised with the use of coxibs,[3–7] but several meta-analyses indicated that both coxibs and some tNSAIDs might be associated with an increased risk of CV thrombotic events.[8–12] However, evidence on the risk of CV events associated with the use of individual NSAIDs is scarce, especially for ischemic stroke (IS).

This study is part of the EU-funded project “Safety of Non-Steroidal Anti-Inflammatory Drugs” (SOS), in which risks associated with individual NSAID use were assessed based on data from six healthcare databases in four European countries. Due to the large study population, the risks could also be evaluated for less frequently used and previously not evaluated individual NSAIDs. Additionally, the effects of duration of use, risk factors of stroke, and co-medication were investigated.

## Methods

Data for this study were obtained from six healthcare databases from Germany, Italy, the Netherlands, and the United Kingdom covering over 32 million people (details in Table 1).[13]

**Table 1:** Characteristics of the participating databases.

Database	GePaRD	IPCI	PHARMO	SISR	OSSIFF	THIN
**Country**	Germany	Netherlands	Netherlands	Italy	Italy	United Kingdom
**Type of Database**	Claims database	General practice database	Record linkage system	National Health Services registry (claims)	National Health Services registry (claims)	General practice database
**Study period**	2005–2009	1999–2011	1999–2008	2000–2009	2002–2009	1999–2008
**Population**	13.7 million	600,000	2.2 million	7.5 million	2.9 million	4.8 million
**New user cohort**	2,139,681	180,988	831,662	2,274,619	1,104,880	1,376,953
**Coding system for diagnoses**	ICD-10-GM	ICPC and free text	ICD-9-CM	ICD-9-CM	ICD-9-CM	READ
**Outpatient hospital diagnoses**	Available	Available, as free text or codes	Available	Available	Available	Available
**Hospital discharge diagnoses**	Available	Available, as free text or codes	Available	Available	Available	Available
**Diagnostic procedures**	Available	Not available	Available	Available	Available	Available
**Laboratory tests**	Available ordering of the test	Available	Available, for a subset	Available	Available	Available
**Coding system for drugs**	ATC	ATC	ATC	ATC	ATC	BNF/ Multilex
**Date of prescription/dispensing**	Available	Available	Available	Available	Available	Available
**Dosing regimen**	Not available	Available	Available	Not available	Not available	Available
**Drug quantity**	Available	Available	Available	Available	Available	Available

GePaRD: German Pharmacoepidemiological Research Database; IPCI: Integrated Primary Care Information database; SISR: Sistema Informativo Sanitario Regionale database; THIN: The Health Improvement Network database; ICD-10-GM: International Classification of Diseases, 10^th^ Revision German Modification; ICD-9-CM: International Classification of Diseases, 9^th^ Revision Clinical Modification; ICPC: International Classification for Primary Care; ATC: Anatomical Therapeutic Chemical classification; BNF: British National Formulary.

This analysis was exclusively based on routinely collected anonymized data and adhered to the European Commission’s Directive 95/46/EC for data protection. The study protocol was approved by the databases’ scientific and ethical advisory boards or regulatory authorities, where applicable, i.e. by the German Federal Insurance Office and the Senator for Labor, Women, Health, Youth and Social Affairs (GePaRD), the IPCI scientific advisory board and the THIN Scientific Review Committee (SRC). No extra approval for data use was needed for PHARMO, SISR and OSSIFF.

Due to time varying nature of drug exposure, the large amount of potentially time varying confounders, the size of the cohort and the log duration of follow-up we performed a case-control study nested in a cohort of new NSAID users.[14] In these situations, a nested case control is computationally more efficient than a Cox analysis based on the full cohort and the estimated odds ratios are unbiased estimators of incidence rate ratios with little or no loss in precision.[15, 16] The study period started on July 1, 1999, and ended on December 31, 2010.

Individuals were included if ≥ 18 years who had (i) ≥ 12 months of continuous enrolment in the database before initial prescription/dispensing of an NSAID (ATC code M01A), (ii) no use of any NSAID in these 12 months, and (iii) no diagnosis of malignant cancer except non-melanoma skin cancer during these 12 months. Cohort entry was the date of the first NSAID prescription/dispensing. Cohort exit was defined as the first of the following: (i) end of study period, (ii) occurrence of IS, (iii) end or interruption of membership, (iv) diagnosis of malignant cancer except non-melanoma skin cancer, or (v) death.

Acute IS was defined as cerebral infarction or stroke of ischemic origin or stroke not specified as hemorrhagic or subarachnoid bleeding and operationalized as a discharge diagnosis with the respective code. Despite differing coding systems between databases, the outcome definition was harmonized by mapping the disease concept included in the definition using the Unified Medical Language System.[17]

The index date for cases was defined as the date of the first diagnosis of or first hospitalization for IS after cohort entry. For each case, up to 100 sex- and age-matched controls (± 1 year) were selected within each database. The index day for controls was the date of the event of the respective case. Thus, controls were implicitly matched on database and date. Cases were eligible to be selected as a control before their index day.

Exposure status at the index day was categorized as follows: (i) *current*: If the drug supply overlapped the index date or ended within the 14-day period before the index date, (ii) *recent*: If the supply ended between 15 and 183 days before the index date, or (iii) *past*: If the supply ended more than 183 days before the index date. NSAIDs were classified into coxibs (celecoxib, etoricoxib, lumiracoxib, rofecoxib and valdecoxib) and traditional, i.e. non-coxib NSAIDs.

The prescribed duration was used if recorded in the database. If the duration was not recorded or was generally not available, the defined daily dose (DDD) was used to estimate the duration of a prescription assuming the use of one DDD per day.

Duration of continuous use was categorized as: (i) < 7 days, (ii) 7 ≤ duration < 30 days, (iii) 30 ≤ duration < 90 days, or (iv) ≥ 90 days. To estimate the duration of continuous use, prescriptions were considered consecutive if the gap between the end of the previous and the following prescription was less than 14 days.

Sex, age, lifestyle information, co-morbidity, and use of drugs were considered as potential confounders (for detailed definitions and specifications see S1 Methods). Harmonization of confounders was performed similarly to outcome harmonization.

Potential confounders were assessed in the twelve-month period before cohort entry. Drugs with a potential pharmacological interaction with NSAIDs or confounding drugs were assessed within 90 days or, for acute treatments, 30 days before the index date.

To estimate the risk of IS associated with current use of individual NSAIDs compared to past use of any NSAID as reference, matched odds ratios (OR) and matched ORs additionally adjusted for potential confounders and their 95% confidence intervals (CIs) were calculated using conditional logistic regression. We used past users instead of never users of NSAIDs as reference group to prevent confounding by indication which was one of the main problems in previous published studies.[11] Important risk factors for IS, i.e. prior history of stroke, transient ischemic attack (TIA), acute myocardial infarction, heart failure, atrial fibrillation and flutter, diabetes mellitus (DM), hyperlipidemia, use of angiotensin-converting-enzyme (ACE) inhibitors/ angiotensin (AT) II antagonists, calcium channel blockers, beta-blockers and other antihypertensive drugs, and smoking and concurrent use of lipid modifying agents, aspirin, anticoagulants, and platelet aggregation inhibitors, were a-priori included in the model. Other potential confounders were tested in a backward elimination process to avoid problems with zero cells in the planned stratified analyses. Analyses were first performed for each database separately. Then, case-control sets from all databases were pooled and analyzed together. Further analyses were performed to assess the effect of duration of continuous use. Additionally, analyses were stratified by sex, age, risk factors of stroke and selected co-medication.

## Results

Overall, 4,593,778 new NSAID users were included. During the study period, 49,170 cases of IS were observed of which 49,118 could be matched to controls. Half of the cases occurred in females (50.3%). Cases were on average 72.7 years old (standard deviation 12.17), controls were slightly younger with a mean of 71.8 years (11.80). The distribution of potential confounders and respective unadjusted and adjusted matched ORs are displayed in Table 2.

**Table 2:** Characteristics of cases with ischemic stroke and matched controls and matched and additionally adjusted ORs of stroke for the respective characteristcs.

	Cases N = 49,118	Controls^§^ N = 4,544,608	Matched OR for IS (95% CI)	Additionally adjusted OR for IS (95% CI)
Female	24,685 (50.3%)	2,283,511 (50.3%)		
Age in years (Mean (SD))	72.7 (12.17)	71.8 (11.80)		
Follow-up in days	1079.5 (854.52)	1072.6 (845.41)		
Prior history of*				
acute myocardial infarction^#^	1,078 (2.2%)	62,898 (1.4%)	1.62 (1.52–1.72)	0.96(0.90–1.03)
ischemic heart disease^##^	3,698 (7.5%)	241,757 (5.3%)	1.49 (1.44–1.55)	^§§^
other cardiovascular disease^##^	4,034 (8.2%)	291,613 (6.4%)	1.33 (1.28–1.38)	^§§^
heart failure^#^	2,530 (5.2%)	153,978 (3.4%)	1.51 (1.44–1.57)	0.99 (0.95–1.04)
peripheral arterial diseases	1,111 (2.2%)	63,841 (1.4%)		^§§^
atrial fibrillation and flutter^#^	1,720 (3.5%)	80,948 (1.8%)	1.99 (1.89–2.09)	1.41 (1.33–1.48)
diabetes mellitus^#^	7,115 (14.5%)	365,586 (8.0%)	1.99 (1.94–2.04)	1.63 (1.58–1.67)
hyperlipidemia^#^	8,709 (17.7%)	678,741 (14.9%)	1.26 (1.23–1.29)	0.84 (0.82–0.87)
hypertension^#^	2,788 (5.7%)	178,715 (3.9%)	1.46 (1.41–1.52)	1.13 (1.09–1.18)
alcohol abuse	1,724 (3.5%)	129,096 (2.8%)		^§§^
obesity	2,785 (5.7%)	205,615 (4.5%)		^§§^
smoking^#^	1,340 (2.7%)	94,149 (2.1%)	1.33 (1.25–1.40)	1.27 (1.20–1.35)
stroke^#^	2,396 (4.9%)	60,329 (1.3%)	4.03 (3.85–4.22)	2.64 (2.52–2.77)
transient ischemic attack^#^	909 (1.9%)	29,830 (0.7%)	2.71 (2.53–2.90)	1.55 (1.44–1.66)
other cerebrovascular disease	1,616 (3.3%)	90,498 (2.0%)		^§§^
migraine	367 (0.8%)	33,998 (0.8%)		^§§^
osteoarthritis^##^	5,198 (10.6%)	439,413 (9.7%)	1.04 (1.01–1.07)	^§§^
RA and inflammatory polyarthritis	488 (1.0%)	38,115 (0.8%)		^§§^
chronic liver disease	1,341 (2.7%)	103,817 (2.3%)		^§§^
kidney failure	764 (1.6%)	40,788 (0.9%)		^§§^
coagulation disorders	398 (0.8%)	24,424 (0.5%)		^§§^
Prior use of drugs*				^§§^
ACE inhibitor/AT II antagonists^#^	13,801 (28.1%)	983,548 (21.6%)	1.43 (1.40–1.46)	1.10 (1.08–1.13)
calcium channel blockers^#^	11,840 (24.1%)	815,138 (17.9%)	1.46 (1.43–1.49)	1.16 (1.13–1.18)
beta blockers^#^	10,321 (21.0%)	720,906 (15.9%)	1.42 (1.39–1.46)	1.14 (1.11–1.16)
cardiac glycosides	2,868 (5.8%)	149,091 (3.3%)		^§§^
combinations and other hypertensive drugs^#^	7,422 (15.1%)	610,368 (13.4%)	1.21 (1.18–1.24)	1.05 (1.03–1.08)
drugs for the treatment of rheumatoid arthritis	2,016 (4.1%)	151,590 (3.3%)		^§§^
*Concurrent use of*				^§§^
diuretics**^,^^##^	12,913 (26.3%)	862,943 (19.0%)	1.45 (1.42–1.48)	1.12 (1.09–1.14)
nitrates**^,^^##^	5,508 (11.2%)	335,838 (7.4%)	1.54 (1.49–1.58)	^§§^
lipid modifying agents**^,^ ^#^	12,037 (24.5%)	868,336 (19.1%)	1.42 (1.39–1.45)	0.98 (0.95–1.01)
Cyp2C9 inhibitor**	879 (1.8%)	69,929 (1.5%)		^§§^
Cyp2C9 inhibitor***	144 (0.3%)	10,072 (0.2%)		^§§^
aspirin**^,^ ^#^	15,256 (31.1%)	851,538 (18.7%)	1.98 (1.94–2.02)	1.80 (1.76–1.84)
anticoagulants**^,^ ^#^	3,302 (6.7%)	221,222 (4.9%)	1.41 (1.36–1.46)	1.32 (1.27–1.37)
platelet aggregation inhibitor**, #	4,778 (9.7%)	164,273 (3.6%)	2.88 (2.80–2.97)	2.52 (2.44–2.60)
aspirin***^,^ ^#^	408 (0.8%)	13,642 (0.3%)	2.57 (2.32–2.84)	2.27 (2.06–2.52)
glucocorticoids**	2,594 (5.3%)	195,124 (4.3%)		^§§^
postmenopausal hormone therapy**	1,046 (2.1%)	105,842 (2.3%)		^§§^
oral contraceptives**	145 (0.3%)	9,911 (0.2%)		^§§^

OR: odds ratio; CI: confidence interval; SD: standard deviation; RA: Rheumatoid arthritis; ACE: angiotensin-converting-enzyme; AT: angiotensin

^§^ up to 100 controls matched on database, sex, age at cohort entry and index date by risk set sampling

^§§^ not included in the final model (eliminated in the backward selection process)

* assessed in the 12 months before cohort entry

** assessed in the 90 days before index date

*** assessed in the 30 days before index date

^#^ a-priori confounder

^##^ selected other confounder

**Fig 1:**
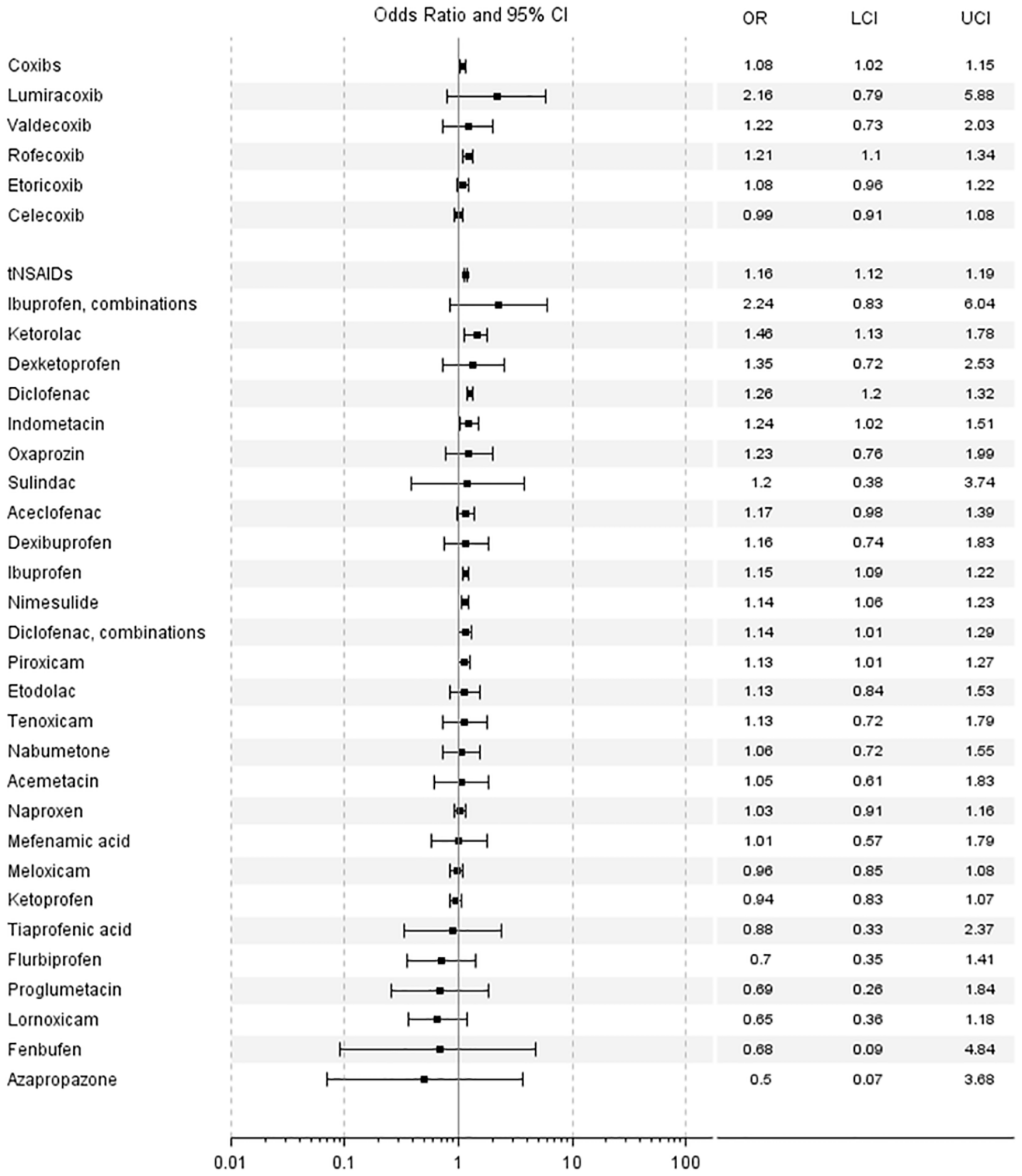
Risk of ischemic stroke associated with the use of NSAIDs: Odds rations (ORs) with 95% confidenceintervals (CIs), lower limit (LCI) and upper limit (UCI).

Matched and confounder-adjusted ORs for current use of each NSAID compared to past use of any NSAID are displayed in Table 3 and Fig 1.

**Table 3:** Risk of ischemic stroke associated with the use of NSAIDs.

	Cases N = 49,118	Controls N = 4,544,608	Matched OR (95% CI)	Additionally adjusted OR* (95% CI)
Past use of any NSAID (reference)			1.0	1.0
Recent use of any NSAID	14,160 (28.8%)	1,307,887 (28.8%)	1.04 (1.01–1.06)	**1.05 (1.02–1.07)**
Current use of Coxibs	1,264 (2.6%)	109,807 (2.4%)	1.07 (1.01–1.13)	**1.08 (1.02–1.15**)
Current use of				
Lumiracoxib	4 (0.0%)	159 (0.0%)	2.32 (0.86–6.29)	2.16 (0.79–5.88)
Valdecoxib	15 (0.0%)	1116 (0.0%)	1.28 (0.77–2.13)	1.22 (0.73–2.03)
Rofecoxib	440 (0.9%)	33,702 (0.7%)	1.21 (1.09–1.33)	**1.21 (1.10–1.34)**
Etoricoxib	261 (0.5%)	22,977 (0.5%)	1.06 (0.94–1.20)	1.08 (0.96–1.22)
Celecoxib	557 (1.1%)	52,816 (1.2%)	0.97 (0.89–1.06)	0.99 (0.91–1.08)
Current use of tNSAIDs	5,814(11.8%)	477,352 (10.5%)	1.16 (1.13–1.20)	**1.16 (1.12–1.19)**
Current use of				
Ibuprofen, combinations	4 (0.0%)	173 (0.0%)	2.12 (0.78–5.71)	2.24 (0.83–6.04)
Ketorolac	97 (0.2%)	5,854 (0.1%)	1.60 (1.31–1.96)	**1.46 (1.19–1.78)**
Dexketoprofen	10 (0.0%)	659 (0.0%)	1.46 (0.78–2.72)	1.35 (0.72–2.53)
Diclofenac	2,044 (4.2%)	158,867 (3.5%)	1.22 (1.17–1.28)	**1.26 (1.20–1.32)**
Indometacin	102 (0.2%)	7,425 (0.2%)	1.27 (1.05–1.55)	**1.24 (1.02–1.51)**
Oxaprozin	17 (0.0%)	1,384 (0.0%)	1.20 (0.75–1.94)	1.23 (0.76–1.99)
Sulindac	3 (0.0%)	216 (0.0%)	1.21 (0.39–3.79)	1.20 (0.38–3.74)
Aceclofenac	128 (0.3%)	10,822 (0.2%)	1.14 (0.96–1.36)	1.17 (0.98–1.39)
Dexibuprofen	19 (0.0%)	1,586 (0.0%)	1.13 (0.72–1.78)	1.16 (0.74–1.83)
Ibuprofen	1,272 (2.6%)	99,409 (2.2%)	1.13 (1.07–1.20)	**1.15 (1.09–1.22)**
Nimesulide	839 (1.7%)	69,534 (1.5%)	1.19 (1.11–1.28)	**1.14 (1.06–1.23)**
Diclofenac, combinations	259 (0.5%)	20,114 (0.4%)	1.11 (0.98–1.25)	**1.14 (1.01–1.29)**
Piroxicam	320 (0.7%)	27,916 (0.6%)	1.11 (0.99–1.24)	**1.13 (1.01–1.27)**
Etodolac	43 (0.1%)	3,465 (0.1%)	1.10 (0.82–1.49)	1.13 (0.84–1.53)
Tenoxicam	19 (0.0%)	1,686 (0.0%)	1.08 (0.69–1.71)	1.13 (0.72–1.79)
Nabumetone	27 (0.1%)	2,325 (0.1%)	1.05 (0.72–1.53)	1.06 (0.72–1.55)
Acemetacin	13 (0.0%)	1,075 (0.0%)	1.10 (0.63–1.90)	1.05 (0.61–1.83)
Naproxen	273 (0.6%)	24,334 (0.5%)	1.01 (0.90–1.14)	1.03 (0.91–1.16)
Mefenamic acid	12 (0.0%)	1109 (0.0%)	1.00 (0.57–1.78)	1.01 (0.57–1.79)
Meloxicam	286 (0.6%)	27,123 (0.6%)	0.94 (0.83–1.05)	0.96 (0.85–1.08)
Ketoprofen	239 (0.5%)	24,671 (0.5%)	0.94 (0.83–1.07)	0.94 (0.83–1.07)
Tiaprofenic acid	4 (0.0%)	437 (0.0%)	0.83 (0.31–2.24)	0.88 (0.33–2.37)
Flurbiprofen	8 (0.0%)	1116 (0.0%)	0.68 (0.34–1.37)	0.70 (0.35–1.41)
Proglumetacin	4 (0.0%)	577 (0.0%)	0.67 (0.25–1.80)	0.69 (0.26–1.84)
Fenbufen	1 (0.0%)	164 (0.0%)	0.55 (0.08–3.93)	0.68 (0.09–4.84)
Lornoxicam	11 (0.0%)	1632 (0.0%)	0.65 (0.36–1.18)	0.65 (0.36–1.18)
Azapropazone	1 (0.0%)	178 (0.0%)	0.51 (0.07–3.64)	0.5 (0.07–3.68)

*Adjusted for prior history of acute myocardial infarction, heart failure, atrial fibrillation and flutter, diabetes mellitus, hyperlipidemia, hypertension, smoking, stroke and transient ischemic attack and prior use of ACE inhibitor/AT II antagonists, calcium channel blockers, beta blockers, cardiac glycosides, combinations and other hypertensive drugs and concurrent use of diuretics, lipid modifying drugs, aspirin, anticoagulants and platelet aggregation inhibitors.

CI: Confidence interval; OR: Odds ratio; NSAID: non-steroid anti-inflammatory drug; Coxib: COX-2 selective inhibitor; tNSAID: traditional non-steroidal anti-inflammatory drug

Use of coxibs (1.08, 95%-CI 1.02–1.15) and use of tNSAIDs (1.16, 1.12–1.19) were associated with an increased risk of IS.

Compared to past use of any NSAID, an increased risk of IS was seen for current use of rofecoxib (1.21, 1.10–1.34), valdecoxib (1.22, 0.73–2.03) and lumiracoxib (2.16, 0.79–5.88). However, for valdecoxib and lumiracoxib, the 95%-CI included the null value due to the low number of exposed subjects.

Among the tNSAIDs, the highest risk was seen for current use of ketorolac (1.46, 1.19–1.78), but also current use of diclofenac (1.26, 1.20–1.32), indomethacin (1.24, 1.02–1.51), ibuprofen (1.15, 1.09–1.22), nimesulide (1.14, 1.06–1.23), diclofenac with misoprostol (1.14, 1.01–1.29), and piroxicam (1.13, 1.01–1.27) was associated with an increased risk of IS. Naproxen (1.03, 0.91–1.16), meloxicam 0.96 (0.85–1.08), and ketoprofen (0.94, 0.83–1.07) showed no elevated risk. The same was true for use of some other, more rarely used NSAIDs.

Results of the analysis of the effect of the duration of continuous use are shown in S1 Table. Already short term use of NSAIDs was associated with an increased risk of IS. However, case numbers were too small to compare individual NSAIDs and to determine whether risks increased with longer use.

**Fig 2:**
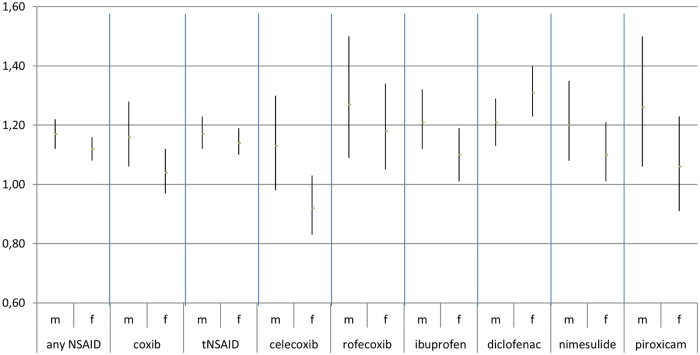
Stratification by sex. Adjusted odds ratios with 95% confidence intervals stratified by sex (m = males, f = females).

**Fig 3:**
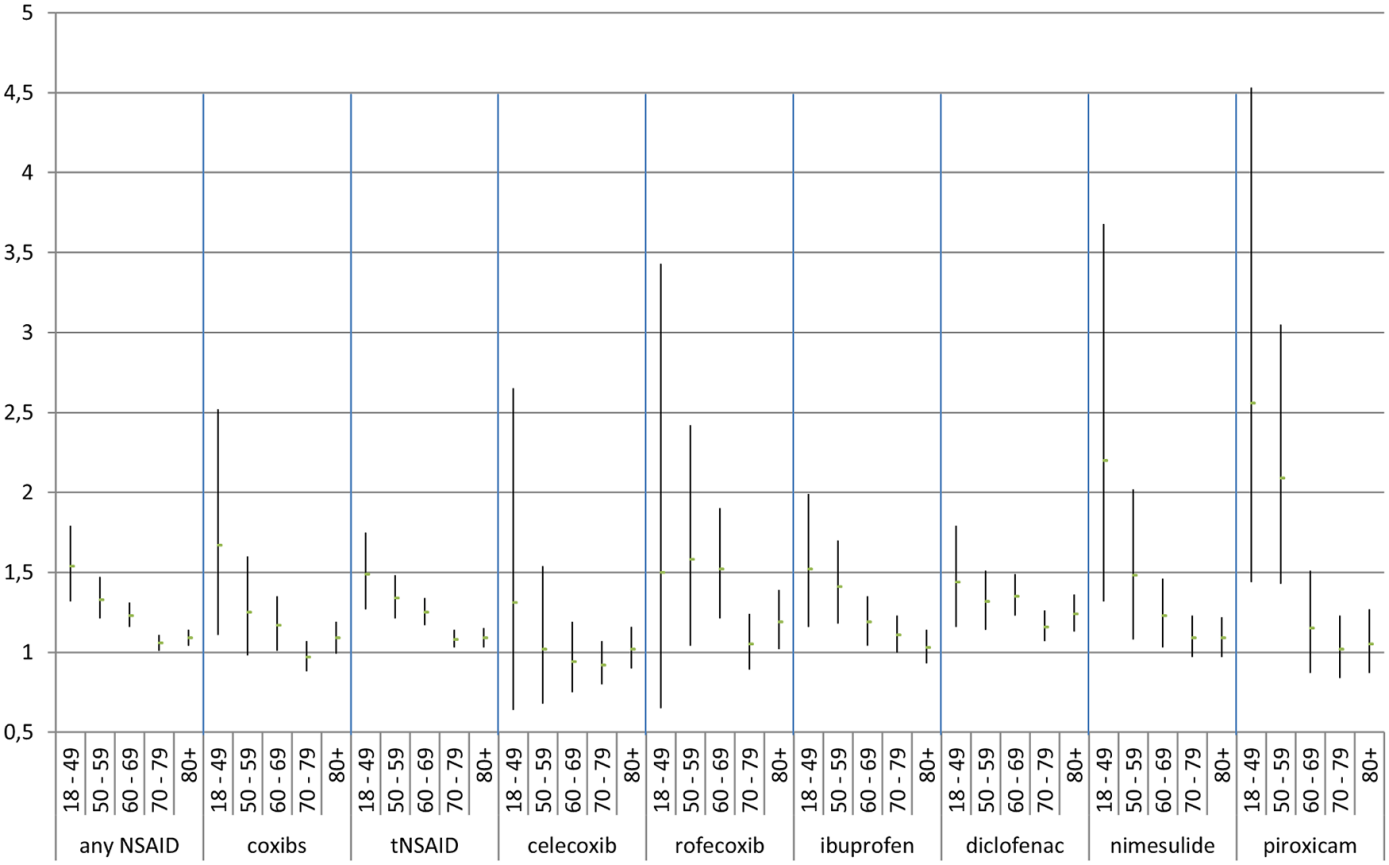
Stratification by age. Adjusted odds ratios with 95% confidence intervals stratified by age groups.

**Fig 4:**
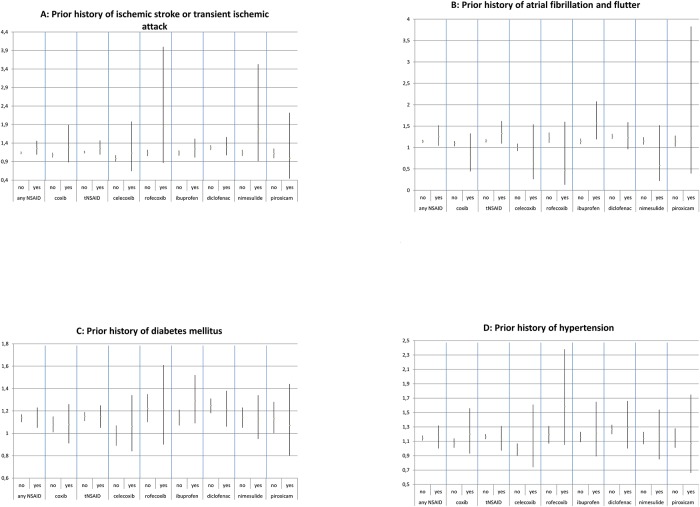
Stratification by risk factors for stroke. Adjusted odds ratios with 95% confidence intervals stratified by risk factors of stroke. (A) Prior history of ischemic stroke or transient ischemic attack. (B) Prior history of atrial fibrillation and flutter. (C) Prior history of diabetes mellitus. (D) Prior history of hypertension.

**Fig 5:**
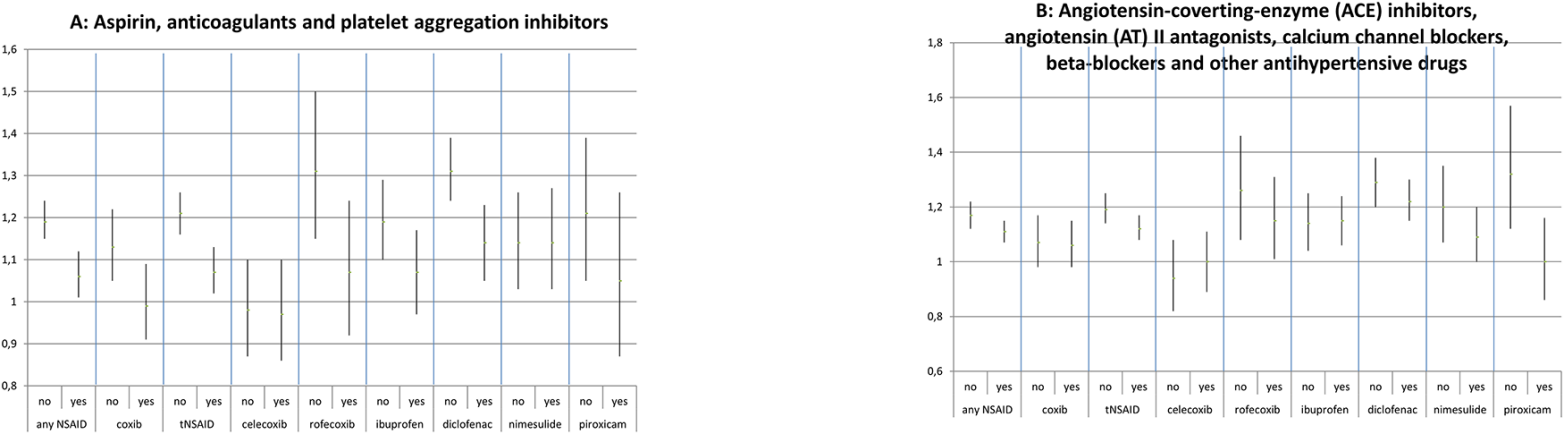
Stratification by prior use of medication. Adjusted odds ratios with 95% confidence intervals stratified by prior use of medication. (A) Aspirin, anticoagulants, and platelet aggregation inhibitors. (B) Angiotensin-coverting-enzyme (ACE) inhibitors, angiotensin (AT) II antagonists, calcium channel blockers, beta-blockers, and other antihypertensive drugs.

Risk estimates were higher in males for the use of any NSAID, coxibs, tNSAIDs, and all examined individual NSAIDs with the exception of diclofenac (Fig 2). However, CIs overlapped. For all NSAIDs, the effect on IS risk seems somewhat stronger among younger people than in older people. (Fig 3). Patients with a prior history of IS or TIA were at a higher risk of IS than patients without such a history when using any NSAID, coxibs, tNSAIDs, and all examined individual NSAIDs except diclofenac and piroxicam (Fig 4). Concomitant use of aspirin, anticoagulants, platelet aggregation inhibitors, and CV medication appears to lower the risk of IS associated with NSAIDs (Fig 5).

## Discussion

This multi-national study evaluated 49,170 IS cases in a cohort of more than 4.5 million new NSAID users. To date, this is the largest study examining the association between IS and use of individual NSAIDs. The study size and the heterogeneity in prescribing patterns across the involved European countries allowed to estimate the risks for 32 individual NSAIDs in real life practice and to examine the effect of risk factors of stroke and relevant co-medications.

Current use of coxibs and tNSAIDs were both associated with an increased risk of stroke compared to past use. However, this risk varied across individual NSAIDs. The highest risk estimate was seen for ketorolac, a tNSAID widely used in Italy. The most frequently used NSAID diclofenac was associated with a 25% increased risk of IS, which was comparable to the risk associated with rofecoxib and indomethacin. Ibuprofen, nimesulide, and piroxicam were associated with an increased risk of about 15%. Current use of meloxicam, ketoprofen, and celecoxib and use of some other, more rarely used NSAIDs did not show an increased risk.

A meta-analysis based on RCTs by the Coxib and traditional NSAID Trialists’ (CNT) Collaboration[12] provided risk estimates for diclofenac, ibuprofen, naproxen, and coxibs that are consistent with our findings (naproxen, coxibs) or slightly lower (ibuprofen, diclofenac). In the network meta-analysis by Trelle et al.,[8] risk estimates for diclofenac, ibuprofen, naproxen, celecoxib, etoricoxib, and rofecoxib were considerably higher, except for rofecoxib. A meta-analysis based on observational studies including more than 10,000 patients with IS[11] yielded risk estimates for naproxen, ibuprofen, diclofenac, and celecoxib in line with our estimates. Interpretation of the observed differences between our findings and the literature is hampered by different designs (e.g., regarding comparator group) and–for the observational studies–methodological issues (e.g., inclusion of prevalent users) of the included single studies.

This study is the first to provide conclusive risk estimates for some less frequently used NSAIDs such as nimesulide, piroxicam, meloxicam, ketoprofen, and indomethacin. Together with the more widely examined NSAIDs, they account for more than 90% of individual NSAIDs used in the four countries.

Our data suggest an early onset of CV effects for most of the NSAIDs. This is different from findings of the Adenomatous Polyp Prevention on Vioxx (APPROVe) study[18] where the incidence rate of thrombotic events of placebo and rofecoxib users was similar for the first 18 months, and an increased risk for rofecoxib was only seen thereafter. However, the study population in this trial was very different from the patient population included in the observational studies as patients with a prior history of stroke and TIA within 2 years were excluded. An early onset of stroke both for tNSAIDs and coxibs was also reported in several observational studies [19–23] and is consistent with the underlying biological mechanisms.

NSAIDs have a broad spectrum of indications: however, they are mostly used by patients with osteoarthritis. For example in a study based on data from the Clinical Practice Research Datalink (CPRD) use was in 66% for osteoarthritis, 21% pain related and in 13% for rheumatoid arthritis.[24] To test whether stroke risk is associated with inflammation but not with NSAIDs themselves, we performed a sub-group analysis including only patients with rheumatoid arthritis and inflammatory polyarthritis or prior use of drugs for the treatment of rheumatoid arthritis. The results of this analysis did not suggest a differential effect among patients with these inflammatory conditions compared to patients in the full cohort. This shows that the observed association (i.e. the observed effects of the individual NSAIDs) cannot be explained by the underlying disease (i.e. inflammation) alone.

Careful NSAID prescribing is recommended in patients with a prior history of IS or TIA, as the associated IS risk of NSAID use seems to be higher in these patients. Concomitant use of aspirin, anticoagulants, and platelet aggregation inhibitors appears to mitigate this risk. We found that the NSAID risk varies by age and sex with higher IS risks observed in younger people and in males except for diclofenac. For some patients with a low baseline risk of IS the absolute risk of IS might not be altered considerably by the choice of NSAID and a small increase might be acceptable if their quality of life improves due to better pain control. However, as IS has serious consequences, we believe that it is worthwhile to avoid even a small increase in risk if a safer alternative–with comparable pain control–is available.

Our study has several limitations, mostly due to the use of secondary data. Lifestyle information (smoking, alcohol, body mass index, physical activity), socio-economic status, and use of over-the counter medication were not—or scarcely—available. However, assessment of co-morbidity and co-medication might capture this information indirectly. This is visible in the ORs, indicating a risk associated with the use of medication that should be protective for IS (e.g., anticoagulants). These ORs do not reflect the effect of these drugs on IS, but rather the risk associated with receiving a prescription for such a drug due to underlying risk factors.

It has been proposed that the CV risk of coxibs results from the imbalance caused by inhibition of COX-2–mediated prostacyclin production without inhibition of COX-1-mediated thromboxane production.[25] More recent research indicates that the CV effects of individual NSAIDs also depend on a complex interaction of pharmacological properties, including duration and extent of platelet inhibition, oxidative stress and renal effects such as volume retention, the extent of blood pressure increase and properties possibly unique to the molecule, as well as pharmacokinetics.[26, 27]

Some misclassification of the outcome is possible, but IS diagnosis has shown good positive predictive values in medical records and claims databases. Further, a validation study in three of the databases included in this study (IPCI, PHARMO and OSSIFF) yielded good concordance between coding and patient charts (unpublished data).

Information on NSAID exposure is precise regarding dispensing time and drug product and recall bias can be ruled out.[28] It is, however, unknown whether the patients took the medication as prescribed which might lead to misclassification of exposure status. As this would usually bias the results towards the null, significant differences as found in this study are still valid.

In all countries at least some of the NSAIDs are also available OTC. However, patients with chronic conditions, such as osteoarthritis and rheumatoid arthritis, get their NSAIDs on prescription. To assess whether OTC use is an important confounder, we performed an analysis including only patients with osteoarthritis, rheumatoid arthritis or inflammatory polyarthritis. The results of this analysis did not suggest a differential effect among the patients with probably very low use of OTC NSAIDs compared to patients in the full cohort.

Residual confounding and especially confounding by indication is always a problem in observational studies. We accounted for this by using a new user cohort, adjusting for many potential confounders in the multivariable analysis and performing sensitivity analyses based on a sub-cohort of patients with osteoarthritis, rheumatoid arthritis and inflammatory polyarthritis or prior use of drugs for the treatment of rheumatoid arthritis, i.e. patients with a similar disease who will probably all take the drug chronically. We also performed several quantitative bias analyses to assess whether the observed effects could be explained by residual confounding.[29] In all scenarios the confounder-exposure association or the confounder-outcome association had to be implausibly strong to nullify the observed associations. Nevertheless, we recognize that residual differences in patient’s baseline characteristics may account for some of the observed variations in relative risk estimates associated with different individual NSAIDs.

The strengths of this study are the size of the source population and the length of follow-up resulting in a number of cases more than four times larger than in previous meta-analyses.[8, 9, 11, 12] The high number of cases allowed to also provide risk estimates for less often used NSAIDs and to examine potential effect modification by risk factors and co-medication. Additionally, we applied a new user design that avoids bias introduced by the fact that patients experiencing side effects are underrepresented in studies based on prevalent users (depletion of susceptibles) and allows the assessment of confounders before start of treatment. In contrast to field studies and due to the secondary nature of the data, no bias is introduced by nonresponse, and coverage of all age groups is complete. In contrast to previous studies, we focused only on IS and did not include hemorrhagic strokes, which have a different pathophysiology and etiology.

In summary, our study shows differences in the association of IS with current use of individual NSAIDs. It indicates a higher risk of NSAID use in patients with a prior history of IS or TIA, in younger patients, and in men. Concomitant use of aspirin, anticoagulants, and platelet aggregation inhibitors appears to mitigate this risk. Both tNSAIDs and coxibs might increase the risk of IS, suggesting that pharmacological properties other than COX-2 selectivity are important. The observed risk estimates might seem small, but as some of the NSAIDs belong to the most widely used drugs worldwide and stroke is one of the leading causes of morbidity and mortality, even an increase in risk of 20% will have a large effect on public health.

## Supporting information

S1 Methods Specification of assessment of lifestyle information, co-morbidity and use of drugs. (DOCX)

S1 Table Risk of ischemic stroke associated with the use of NSAIDs by duration of continuous use. Reference is short duration of use (7–29 days). CI: Confidence interval; OR: Odds ratio.
(DOCX)
