# Supplementary material for: Risk of ischemic stroke and the use of individual non-steroidal anti-inflammatory drugs: A multi-country European database study within the SOS Project
Source: PLoS One. 2018 Sep 19;13(9):e0203362. doi: 10.1371/journal.pone.0203362 (PMC6145581; doi:10.1371/journal.pone.0203362)
Supplement: S1 Methods — (DOCX) [file pone.0203362.s001.docx]

Life Style Information at baseline (assessed in the 12 months before cohort entry)

- Smoking (only in DBs with reliable information on smoking status) – direct information (e.g. note that person is smoker), diagnosis codes and specific drugs
- Alcohol abuse (only in DBs with reliable information on alcohol abuse) – direct information, diagnosis codes and specific drugs
- Obesity – direct information (e.g. BMI), diagnosis codes and specific drugs

Co-Morbidities at baseline (assessed in the 12 months before cohort entry)

- Myocardial infarction (acute, recurrent, silent, complication and old) – diagnosis codes
- Heart failure – diagnosis codes and drugs indicating heart failure (C07AG02)
- Stroke – diagnosis codes
- Transient cerebral ischemic attack (TIA) – diagnosis codes
- Arterial hypertension and hypertensive disease – diagnosis codes
- Chronic ischemic heart disease – diagnosis codes
- Artrial fibrillation and flutter – diagnosis codes
- Peripheral arterial diseases – diagnosis codes
- Other cardiovascular disease
  - Cardiac arrhythmia/conduction disorders and arrest – diagnosis codes or drugs indicating cardiac arrhythmia (C01B)
  - Cardiomyopathies – diagnosis codes
  - Valvular disorders and endocarditis – diagnosis codes
  - Myocarditis and pericarditis – diagnosis codes
  - Arterial embolism and thrombosis – diagnosis codes
- Other cerebrovascular disease – diagnosis codes
- Blood coagulation disorders (specified as increased bleeding risk, thrombosis risk or unspecified) – diagnosis codes
- Kidney failure – diagnosis codes
- Chronic liver diseases – diagnosis codes
- Migraine – diagnosis codes
- Diabetes mellitus – diagnosis codes or drugs indiating diabetes mellitus (A10)
- Hyperlipidemia – diagnosis codes or drugs indicating hyperlipidemia (C10)
- Rheumatoid arthritis and inflammatory polyarthritis – diagnosis codes or specific antirheumatic agents (M01C)
- Osteoarthritis – diagnosis codes

Unspecific Drugs at baseline (assessed in the 12 months before cohort entry)

- Unspecific drugs for rheumatoid arthritis
  - Immunosuppressants (selected subcodes of L04A)
  - Antiklonal antibodies (L01XC02)
  - Chloroquine (P01BA01)
  - Hydrochloroquine (P01BA02)
  - Sulfasalazine (A07EC01)
  - Topical Products for Joint and Muscular Pain (M02)
- ACE inhibitors (C09A) and AT II antagonists (C09C) – unspecific for arterial hypertension, heart failure, coronary heart disease, medication post acute MI
- Calcium channel blockers (C08C, C08D, C08E) – unspecific for arterial hypertension, coronary heart disease, cardiac arrhythmia
- Beta blocking agents (C07A) – unspecific for arterial hypertension, heart failure, coronary heart disease, cardiac arrhythmia, medication post acute MI
- Other hypertensive drugs (C02A, C02B, C02C, C02D, C02K, C09X) and combinations of antihypertensive drugs (C02L, C02N, C07B, C07C, C07D, C07E, C07F, C08G, C09B, C09D, C09X) – unspecific for arterial hypertension
- Cardiac glycosides (C01A) – unspecific for congestive heart failure, cardiac arrhythmia
- Vasodilators used in cardiac diseases exclusive nitrates (C01DB, C01DX) – unspecific for heart failure, coronary heart disease, acute MI

Drugs with Pharmacological Interaction and Confounding Drugs (assessed at index day)

Prescription within 90 days before index day:

- Cyp2C9 inhibiting drugs (long-term use) (without C01AA codes)
- Cyp2C9 inducing drugs (long-term use)
- Glucocorticoids (H02AB, H02B)
- Anticoagulants (B01AA, B01AB, B01AE, B01AX)
- Platelet aggregation inhibitors excl. heparin and ASA (B01AC without ASA codes)
- Aspirin (ASA codes of B01AC)
- Diuretics (C03A, C03B, C03C, C03D, C03E, C03X)
- Nitrates (C01DA)
- Statins (C10AA, C10BA, C10BX)
- Oral contraceptives (G03A, G02BB)
- Postmenopausal hormone therapy (G03C, G03D, G03F, G03HB)

Prescription within 30 days before index day:

- Cyp2C9 inhibiting drugs (short-term use)
- Cyp2C9 inducing drugs (short-term use)
- Antithrombotics- Enzymes (B01AD)
- Aspirin (selected subcodes of N02BA, A01AD, M01BA)
