# Supplementary material for: Risk of ischemic stroke and the use of individual non-steroidal anti-inflammatory drugs: A multi-country European database study within the SOS Project
Source: PLoS One. 2018 Sep 19;13(9):e0203362. doi: 10.1371/journal.pone.0203362 (PMC6145581; doi:10.1371/journal.pone.0203362)
Supplement: S1 Table — Reference is short duration of use (7–29 days). CI: Confidence interval; OR: Odds ratio. (DOCX) [file pone.0203362.s002.docx]

|  | Duration | | | | | |
| --- | --- | --- | --- | --- | --- | --- |
|  | Very short (0-6 days) | | Medium (30-89 days) | | Long ( ≥ 90 days) | |
|  | Cases/Controls | OR (95% CI) | Cases/Controls | OR (95% CI) | Cases/Controls | OR (95% CI) |
| Current Use of Diclofenac | 419/24,412 | **1.41** (1.21-1.63) | 435/34,922 | 1.03 (0.89-1.19) | 224/15,645 | **1.24** (1.02-1.52) |
| Current Use of Piroxicam | 99/6,874 | 1.32 (0.89-1.97) | 60/6,138 | 0.83 (0.50-1.39) | 13/1,189 | 1.93 (0.54-6.97) |
| Current Use of Meloxicam | 29/2,597 | 0.90 (0.36-2.26) | 93/8,578 | 1.03 (0.59-1.82) | 67/6,411 | 2.16 (0.97-4.80) |
| Current Use of Ibuprofen | 231/14,924 | **1.31** (1.08-1.59) | 228/18,178 | 0.97 (0.79-1.18) | 152/8,500 | 1.17 (0.89-1.54) |
| Current Use of Naproxen | 23/2,269 | 1.30 (0.55-3.11) | 87/7,206 | 1.67 (0.91-3.08) | 53/4,406 | 1.00 (0.46-2.18) |
| Current Use of Ketoprofen | 80/6,762 | 1.23 (0.79-1.94) | 40/3,678 | 0.91 (0.49-1.68) | 13/1,413 | 1.30 (0.47-3.64) |
| Current Use of Celecoxib | 52/4,648 | 1.19 (0.77-1.83) | 163/15,988 | 1.08 (0.80-1.44) | 123/10,398 | 1.04 (0.72-1.51) |
| Current Use of Rofecoxib | 44/3,003 | 1.47 (0.97-2.23) | 128/10,362 | 0.99 (0.73-1.34) | 91/6,536 | 1.03 (0.70-1.50) |
| Current Use of Etoricoxib | 32/1,855 | **2.33** (1.04-5.24) | 73/6,715 | **1.94** (1.01-3.74) | 40/3,230 | 1.89 (0.78-4.56) |
| Current Use of Nimesulide | 143/11,231 | 1.08 (0.86-1.35) | 176/12,050 | 1.21 (0.98-1.50) | 16/776 | 1.30 (0.71-2.40) |
